# Prevalence and correlates of mental health problems among different occupations of medical workers during COVID-19 outbreak in China

**DOI:** 10.3389/fpubh.2024.1353608

**Published:** 2024-04-04

**Authors:** Qiuping Yan, Xiaofei Hou, Tingting Zhang, Huifang Yin, Bing Han, Chao Ma, Ying Wang, Hongguang Chen, Jing Wen, Yueqin Huang, Rongmeng Jiang, Zhaorui Liu, Guangming Xu

**Affiliations:** ^1^Tianjin Anding Hospital, Tianjin University, Tianjin, China; ^2^Peking University Sixth Hospital, Peking University, Beijing, China; ^3^Beijing Ditan Hospital, Capital Medical University, Beijing, China

**Keywords:** mental health, medical workers, prevalence, COVID-19, China

## Abstract

**Background:**

Health workers involved in the fight to prevent the COVID-19 outbreak were exposed to hazards. Detailed information on mental health problems in different medical occupations is crucial. To examined the prevalence of mental health issues in three medical occupations as well as the relationships between mental health problems and correlates in each occupation.

**Methods:**

This study utilizing the Questionnaire Star program was conducted among medical workers working at medical institutions in China from February 17 to 24, 2020. The Self-Reporting Questionnaire (SRQ-20), the Zung Self-rating Anxiety Scale (SAS), and the Zung Self-rating Depression Scale (SDS) were used to assess mental health problems.

**Results:**

The prevalence of any mental health problems in the three occupations was 43.6, 34.6, and 32.9% for nurses, paramedical workers (PMWs), and doctors, respectively. Three occupations shared some correlates, such as being overworked, not having enough time to rest, support from colleagues, and previous mental health status. There were specific factors for each occupation. For doctors, age, educational level, living status, support from family, and previous physical status were related factors in mental health problems. Working in a designated hospital for treating COVID-19, having COVID-19 event exposures, and receiving support from family were associated with the mental health problems of the nurses. PMWs’ mental health problems was linked to educational level and care from supervisors or heads of department.

**Conclusion:**

Different medical occupations have distinct impacts on mental health issues. Policy makers and mental health professionals working to prepare for potential disease outbreaks should be aware of multiple factors in different occupations.

## 1 Introduction

In December 2019, the outbreak of COVID-19, a novel coronavirus, which originated in Wuhan, Hubei Province, China. This led to an outbreak of pneumonia that quickly spread over the whole nation, posing significant risks to public health and garnering global attention. Nearly all medical personnel across China were engaged in preventing and controlling COVID-19 infection (1). They were organized to manage and treat patients with COVID-19, and they were exposed to a high risk of infection. By February 11, 2020, 1,716 healthcare workers in mainland China were confirmed to be infected with COVID-19, accounting for 3.8% of confirmed cases; among these healthcare workers, 14.8% of the confirmed cases were diagnosed as severe or critical cases, and 5 cases died (2). Medical workers were undergoing high stress from COVID-19, especially those in the frontline to contact with suspected or confirmed cases (3). At the early stage of COVID-19 outbreak, there were no effective treatments or vaccines for COVID-19 (4). And the working conditions of medical workers has been altered during the COVID-19 period, they had to treat more patients, prolong working hours, and face a high risk of infection any time (5). The uncertainty and deviating from usual working practices may lead to some physical and mental health hazards, including but not limited to social discrimination, psychological strain, fatigue, and occupational exhaustion (6). Studies indicated that those medical health workers had significant prevalence rates of symptoms of depression, anxiety, insomnia, distress (7), somatization, and obsessive-compulsive symptoms (8), and reported that experiencing burnout and acute post-traumatic symptoms to a significant degree (9). One cross-sectional survey in China involving 1,257 medical personnel during the COVID-19 epidemic showed that the prevalence of symptoms of depression was found to be 50.4%, while anxiety was reported by 44.6% of participants. Insomnia was noted in 34.0% of the surveyed medical personnel, distress was prevalent in a staggering 71.5% of individuals, and more than 70% experienced psychological distress (7).

Several factors have been identified to impact the mental health of medical workers during the infectious disease outbreak. First, some unique stressors of COVID-19 for healthcare workers, including overwork, inadequate personal protective equipment, fear of personal infection, and separation from family and friends for a long time, are considered high-risk factors for poor mental health (10, 11). In addition, studies conducted during the COVID-19 pandemic showed that individual physical and mental status could be associated with the symptoms of depression and anxiety among healthcare workers (12), and public as well as healthcare workers with pre-existing physical issues and psychological stressors may exacerbate their current health conditions (13). Therefore, we should not downplay the role of previous physical or mental issues of medical workers during their fight against COVID-19. Moreover, social support is also recognized as an essential mental health correlate (14). For example, there is evidence that support from colleagues, leadership, and families could positively affect the medical staff’s mental health during the COVID-19 epidemic (5, 15). Understanding the factors influencing medical workers’ mental health during the COVID-19 pandemic helps improve their health conditions and better respond to future disease outbreaks.

Some studies have shown the psychological impact of infectious disease on medical workers, and these effects differed with respect to occupation (16–19). Two studies found that nurses might be more likely to have a higher presence of mental health problems than doctors and other medical workers (16, 18). In another study, doctors seemed to feel more stress compared to nurses (19). However, a different study found no distinction between nurses and doctors (17). The results could be attributed to the use of multiple instruments and the inclusion of participants from other departments. Consequently, existing research provides insufficient information regarding mental health issues in various professions, which could assist us in delivering customized mental healthcare for healthcare professionals during and following the COVID-19 pandemic. Furthermore, there is limited knowledge regarding the factors linked to mental health issues among medical professionals in various department. While certain psychological interventions have been established to assist medical personnel during the COVID-19 outbreak (6, 20), it is crucial for health authorities to have comprehensive insights into the prevalence of mental health issues among various medical occupations and their associated factors. This knowledge will aid in the allocation of health resources and the development of suitable treatments for medical workers experiencing mental health problems.

The aims of this study are to: (I) examine the prevalence of mental health problems (psychological distress, anxiety, and depression) in different occupations of medical workers; and (II) explore the associations between mental health problems and a range of important correlates in each occupation. Findings from this study may provide a snapshot of the mental health status and psychological burden healthcare staff faced in the early stages of the epidemic and different correlates of mental health problems of different medical occupations. Understanding the mental health status of medical workers at this stage of COVID-19 helps identify the prior population for psychological interventions and develop systematic psychological interventions to improve the mental health of medical staff in the future pandemic.

## 2 Methods

### 2.1 Participants

The target participants in this survey were medical workers working in medical institutions. The medical institutions encompass hospitals, primary healthcare centers, centres for disease management and prevention, as well as maternity and child health care hospitals and sanatoriums. According to their duties, medical workers were divided into three occupations, including doctors, nurses, and paramedical workers (PMW). Paramedical workers included medical technologists, administrators, logisticians, and researchers.

The sample was acquired using a non-probability sampling methodology, and an approach similar to quota sampling was used to calculate the sample size, guaranteeing an adequate representation of respondents from each occupation. Based on a survey using SDS and SAS during the Severe Acute Respiratory Syndrome (SARS) epidemic, the incidence of depression and anxiety in nurse was 21.42 and 25%, respectively (21). So, the lowest prevalence of mental health problems was assumed to be 20% for each occupation. The minimum required sample size (*n*) was calculated as n = μ_α_^2^ p (1 - p)/δ^2^, where μ_α_ = one-sided magnitude of confidence (μ_α_ = 1.96 for α = 0.05), *p* = expected proportion of the outcome of interest. δ = error range (22). To achieve a relative error of 15%, and considering 5% non-response (23), the minimum effective sample size for each occupation would be about 718. It would require about 2,154 participants in total.

### 2.2 Procedures

A cross-sectional epidemiological research was done from February 17 to 24, 2020, to assess mental health problems among medical professionals working at medical institutes in mainland China (23). An online survey utilizing the Questionnaire Star program (Wenjuanxing), a professional online questionnaire editing program, to edit the questionnaire.^1^ After completing the editing, a QR code-a bar code readable by mobile devices that contains the website link to the questionnaire utilized in this research-was generated, and researchers sent a QR code poster to medical workers via WeChat messages. Organizations use WeChat groups to post work announcements; our researchers initially sent the QR code to different WeChat work groups consisting of medical personnel. In addition, 60 department directors were asked to introduce the survey and QR code poster to their medical colleagues to increase the number of participants. The participants can scan the code to access the Questionnaire Star website and complete the questionnaire electronically. All medical personnel who received a QR code poster were eligible to take part in the survey.

Twenty-three small WeChat groups (of 50–60 members each) and five large WeChat groups (of 500 members each) were provided with the QR code. Additionally, the QR code was distributed to 60 department directors from 60 hospitals to increase the number of participants, and these directors were asked to share the code among their staff members. Once the questionnaire was submitted, the data would be stored on the server of Questionnaire Star and could only be downloaded by an authorized data manager.

The Ethical Committee of Beijing Ditan Hospital Capital Medical University (BJDTH) has granted approval for this survey (JINGDILUNKE (2020)-(012)-01). Writing informed consent was not required of any participant in this web-based survey, as our participation was entirely anonymous and voluntary. Participants were permitted to exit or interrupt the survey without providing an explanation for their actions, and the objectives and methods of the survey were stipulated in a clear manner at the beginning of the survey. And after completion of information collection and collation, the electronic version of the database was password-set for the sole use of the research team to strictly protect the security of the data. All investigational procedures were conducted in accordance with the 1964 Helsinki Declaration and its subsequent amendments, as well as the pertinent ethical regulations of the national and internal research committees.

### 2.3 Measurements

#### 2.3.1 Mental health problems

##### 2.3.1.1 Psychological pain

The psychological pain was evaluated by the WHO 20-item Self-Reporting Questionnaire (SRQ-20) (24) was used to assess three mental health issues: psychological pain, anxious symptoms, and depressive symptoms, and the questionnaire has been validated in a Chinese population (25). The SRQ-20 is a self-report questionnaire comprising 20 items suggested by the WHO to identify and assess general mental disorders. Each item is rated as “yes” or “no.” For each “yes” response, the participant receives 1 point, and for each “no” response, the participant receives 0 points. The total score for SRQ-20 ranges from 0 to 20. The SRQ-20 item questions pertain to symptoms related to depression, anxiety, and psychosomatic complaints. The result is defined as SRQ positive when the participant’s overall SRQ-20 score is higher than seven, which indicates the participant is experiencing psychological pain and needs professional assistance (24). The SQR-20 has demonstrated good internal consistency with Cronbach’s α coefficient of more than 0.87 in the Chinese nurse sample (26).

##### 2.3.1.2 Anxious symptoms and depressive symptoms

The Zung Self-Rating Anxiety Scale (SAS) (27) and the Zung Self-Rating Depression Scale (SDS) (28) were employed to evaluate anxious symptoms and depressive symptoms, respectively. Either the SAS or the SDS is a 20-item self-report questionnaire, and participants were instructed to evaluate each item using four-point Likert scale, with responses ranging from 1 (never) to 4 (always). SAS evaluates the severity of anxious symptoms. The thresholds for identifying anxious symptoms were defined as: standard score < 50 no symptom, 50–59 mild, 60–69 moderate, and ≥ 70 severe (29). In the previous study, the Cronbach’ α coefficient of Chinese version of this scale is 0.78 and the split-half reliability is 0.75 (30). SDS assesses the level of depressive symptoms. The cut-off points for depressive symptoms (29) were the same as those for anxious symptoms. The Cronbach’s α coefficient of the Chinese version of SDS is 0.86, and the test–retest reliability (3-week) was 0.83 (31).

In this study, participants were asked to provide their mental health status using the SRQ-20, SAS, and SDS during the past week. The survey was conducted during the most severe of the COVID-19 epidemic, considering the rapidly changing nature of the COVID-19 situation, to investigate specifically the impact on the mental health of medical staff in such a context, the duration of the survey was restricted to a span of 1 week.

#### 2.3.2 Correlates

The following sociodemographics were assessed: age, gender, educational level, marital status, living condition (living with family members most of the time or not), and having a child <18 years old or not.

The working status of the sample was determined by survey respondents’ professional titles, designated institution for COVID-19 treatment, department with high or low risk during the COVID-19 pandemic (We defined a healthcare professional who was employed in any of the four departments listed below—infection, emergency, intensive care unit, or respiratory—could be considered to be in a high-risk department), experience of treating COVID-19 (yes, no), previous experience of treating infectious diseases (yes, no), training experiences of nosocomial infections before and after COVID-19 epidemic and the practice guideline of COVID-19 (yes, no), feel overworked (yes, no), enough time to rest (yes, no), adequate personal protective equipment (PPE, very adequate, partly adequate, not adequate), adequate care from leaders of the unit or department (yes, no), getting colleagues’ adequate support (always, most of the time, hard to say or no), and family’s attitude toward the nature of the current work (totally support, partly support, do not support).

Information about COVID-19 event exposures of participants was obtained by asking two questions: (i) Have you or your relative/friends/colleagues been diagnosed with COVID-19 pneumonia (yes, no)? (ii) Have your relatives/friends/colleagues been quarantined as the suspected case (yes, no)? Participants with a positive answer on any of the two questions were defined as positive COVID-19 event exposures.

The physical and mental health condition prior to the COVID-19 outbreak were also investigated by asking participants about previous physical health and previous mental health (very good, good, just so-so, or poor), respectively.

### 2.4 Statistical analyses

The prevalence of mental health problems was estimated for SRQ-20, SAS, and SDS-positive. Weighting was not considered during analysis as the sample design of the survey was non-probability. The *χ*^2^ test was used to test the prevalence between three occupations. The Poisson regression was used to analyze the prevalence ratios (PR) by controlling age, gender, education, marital status, and profession (32). A participant was defined as having any mental problem if he or she received one positive score on the three scales. In each occupational category, univariate logistic regressions were conducted to analyze the relationships between having any mental problem and correlates, followed by multivariable logistic regression that showed significant univariate associations. PR values in Poisson regression and OR values in logistic regression and their 95% Confidence Intervals (95%CI) were reported. In this study, a *p* value less than 0.05 was considered significant. All statistical analyses were conducted using the Statistical Package for Social Sciences (SPSS, version 22.0; SPSS Inc., Chicago, IL, USA).

## 3 Results

### 3.1 Characteristics of the sample

A sample of 5,495 medical workers submitted the questionnaire, nine invalid responses were excluded. Of the 5,486 medical workers from 471 medical institutions in 31 provinces, 5,380 (98.1%) worked in hospitals, 72 (1.3%) in primary care institutes, 32 (0.6%) in maternal and child health care hospitals and sanatoriums, and 2 at the CDC. About 44.9% of the sample was from Beijing, the capital of China, and only 2.5% was from Hubei, where the largest numbers of COVID-19 patients were located. The sample involved 1,916 (35.0%) doctors, 2,857 (52.0%) nurses, and 713 (13.0%) PMWs. The average ages of the total sample, doctors, nurses, and PMWs were 36.2 ± 9.0 years old, 40.3 ± 8.6 years old, 33.1 ± 8.1 years old, and 37.6 ± 8.9 years old, respectively.

The characteristics of the three occupations are shown in Table 1. There were significant differences among the three occupations in most variables. Nurses had a higher female percentage than doctors and PMWs. More than 94% of participants had experience receiving training about infectious diseases and training of COVID-19. More than 30% of doctors, 23% of nurses, and 28% of PMWs were overworked. More than 25% of medical workers did not have enough time to rest during the COVID-19 outbreak. More than 37% of medical workers did not get enough PPE. More than 36% of medical workers reported they did not receive enough care from their leaders, and about 11% of medical workers did not get enough support from colleagues. About 6% of medical workers could not receive enough support from their families. There were no significant differences among three occupations on the variables about the previous training in treating infectious disease, training of COVID-19, and previous physical health.

**Table 1:** The characteristics of the sample by each occupation (*n*, %).

Variables		Doctors (*N* = 1,916)	Nurses (*N* = 2,857)	PMW (*N* = 713&)
Gender*	Female	1,175 (61.3)	2,737 (95.8)	521 (73.1)
Age*	< 35	551 (28.8)	1,819 (63.7)	289 (40.5)
	35–44	742 (38.7)	696 (24.4)	249 (34.9)
	> 44	623 (32.5)	342 (12)	175 (24.5)
Education level*	College certificate or lower	94 (4.9)	1,180 (41.3)	174 (24.4)
	Bachelor	1,066 (55.6)	1,668 (58.4)	464 (65.1)
	Master or PhD	756 (39.5)	9 (0.3)	75 (10.5)
Marital status*	Single	233 (12.2)	843 (29.5)	117 (16.4)
	Married	1,621 (84.6)	1,934 (67.7)	574 (80.5)
	Divorced/Widowed	62 (3.2)	80 (2.8)	22 (3.1)
Living with family members*	Everyday/Most of the time	1,381 (72.1)	1,994 (69.8)	582 (81.6)
	Seldom/not	535 (27.9)	863 (30.2)	131 (18.4)
Having children younger than 18 years old*	Yes	1,117 (58.3)	1,443 (50.5)	384 (53.9)
	No	799 (41.7)	1,414 (49.5)	329 (46.1)
Designated institution for COVID-19 treatment*	Yes	1,014 (52.9)	1,560 (54.6)	430 (60.3)
	No	902 (47.1)	1,297 (45.4)	283 (39.7)
Department*	High-risk	853 (44.5)	1,277 (44.7)	65 (9.1)
Previous training in treating infectious disease	Yes	1,841 (96.1)	2,730 (95.6)	671 (94.1)
	No	75 (3.9)	127 (4.4)	42 (5.9)
Training of COVID-19	Yes	1,896 (99)	2,837 (99.3)	702 (98.5)
	No	20 (1)	20 (0.7)	11 (1.5)
Professional title*	Primary or lower	508 (26.5)	1,895 (66.3)	349 (48.9)
	Intermediate	674 (35.2)	872 (30.5)	247 (34.6)
	Senior	734 (38.3)	90 (3.2)	117 (16.4)
Previous experience in treating infectious disease*	Yes	1,300 (67.8)	1,281 (44.8)	156 (21.9)
	No	616 (32.2)	1,576 (55.2)	557 (78.1)
Experience of treating COVID-19*	Yes	688 (35.9)	756 (26.5)	91 (12.8)
	No	1,228 (64.1)	2,101 (73.5)	622 (87.2)
Overworked*	Yes	613 (32.0)	678 (23.7)	203 (28.5)
	No	1,303 (68.0)	2,179 (76.3)	510 (71.5)
Enough rest time*	Yes	1,337 (69.8)	2,128 (74.5)	490 (68.7)
	No	579 (30.2)	729 (25.5)	223 (31.2)
Adequate PPE*	Very adequate	301 (15.7)	520 (18.2)	96 (13.5)
	Partly adequate	876 (45.7)	1,279 (44.8)	314 (44.0)
	Not adequate	739 (38.6)	1,058 (37.0)	303 (42.5)
Care from leaders*	Yes	1,015 (53)	1,825 (63.9)	386 (54.1)
	No	901 (47.0)	1,032 (36.1)	327 (45.9)
Colleagues’ support*	Always	882 (46.0)	1,446 (50.6)	334 (50.6)
	Most of the time	808 (42.2)	1,086 (38.0)	284 (39.8)
	Hard to say or no	226 (11.8)	325 (11.4)	95 (13.3)
Family support*	Totally support	1,179 (61.5)	1,781 (62.3)	482 (67.6)
	Partly support	610 (31.8)	847 (29.6)	187 (26.2)
	Do not support	127 (6.6)	229 (8.0)	44 (6.2)
Previous mental health*	Very good	841 (43.9)	1,170 (41.0)	305 (42.8)
	Good	763 (39.8)	1,143 (40.0)	299 (41.9)
	Just so-so or poor	312 (16.3)	544 (19.0)	109 (15.3)
Previous physical health	Very good	735 (38.4)	1,051 (36.8)	277 (38.8)
	Good	804 (42.0)	1,161 (40.6)	291 (40.8)
	Just so-so or poor	377 (19.7)	645 (22.6)	145 (20.3)
COVID-19 event exposures*	Yes	336 (17.5)	229 (8.0)	42 (5.9)
	No	1,580 (82.5)	2,628 (92.0)	671 (94.1)

PMW, paramedical workers; PPE, personal protective equipment. *Significant difference among three occupations (*p* < 0.05) and include 463 medical technologists, 173 administrations, 48 logisticians and 29 researcher.

### 3.2 The prevalence of mental health problems

The prevalence of mental health problems in each occupation is shown in Table 2. The prevalence of psychological pain in three occupations was 16.7% in doctors, 15.2% in nurses, and 13.5% in PMWs, respectively. Nurses had the highest prevalence of anxiety (39.1%; 95%CI: 37.3–30.9%) than those doctors (27.9%; 95%CI: 25.9–29.9%) and PMWs (30.0%; 95%CI: 27.3–34.1%) respectively. The prevalence of depression in nurses (17.4%; 95%CI: 16.0–18.8%) was higher than that in doctors (13.8%; 95%CI: 12.3–15.4%) and PMWs (13.7%; 95%CI: 11.2–16.3%). Regarding the prevalence of any mental health problems, nurses had the highest percentage (43.6%), followed by PMWs (34.6%; 95%CI: 31.1–38.1%), and doctors (32.9%; 95%CI: 30.8–35.0%). There were significant differences among the three occupations in the prevalence of anxiety, depression, and any mental health problems.

**Table 2:** The prevalence of mental health problems in each occupation of healthcare workers in China.

	Doctor (*N* = 1,916)		Nurse (*N* = 2,857)		PMW (*N* = 713)		*χ* ^2^	*p*
	*n*	%(95%CI)	*n*	%(95%CI)	*n*	%(95%CI)		
Psychological pain	320	16.7 (15.0–18.4)	435	15.2 (13.9–16.5)	96	13.5 (11.0–16.0)	4.528	0.104
Anxious symptom	534	27.9 (25.9–29.9)	1,118	39.1 (37.3–40.9)	219	30.0 (27.3–34.1)	68.908	<0.001
Depressive symptom	265	13.8 (12.3–15.4)	498	17.4 (16.0–18.8)	98	13.7 (11.2–16.3)	13.588	0.001
Any symptom	631	32.9 (30.8–35.0)	1,247	43.6 (41.8–45.5)	247	34.6 (31.1–38.1)	60.26	<0.001

The prevalence ratios (PR) between three occupations were illustrated in Table 3. Doctors had significant higher psychological pain than PMWs (PR = 1.30; 95%CI: 1.02–1.65). Nurses had significant higher prevalence of anxiety and depression than doctors and PMWs. Moreover, nurses also had significant higher prevalence of any mental health problems than doctors (PR = 1.23; 95%CI: 1.08–1.41) and PMWs (PR = 1.25; 95%CI: 1.09–1.45).

**Table 3:** Prevalence ratios (95%CI) from a poisson regression for the independent effects of occupation on mental health problems^&^.

Occupations	Psychological pain (*N* = 851)	Anxious symptom (*N* = 1,871)	Depressive symptom (*N* = 861)	Any symptom (*N* = 2,125)
PMW	0.77 (0.61–0.98)*	0.95 (0.74–1.21)	1.02 (0.87–1.21)	0.99 (0.84–1.15)
Nurse	0.90 (0.74–1.09)	1.25 (1.02–1.53)*	1.28 (1.11–1.48)*	1.23 (1.08–1.41)*
Doctor	1	1	1	1
Doctor	1.30 (1.02–1.65)*	1.05 (0.83–1.35)	0.83 (0.83–1.15)	1.02 (0.87–1.19)
Nurse	1.16 (0.93–1.47)	1.32 (1.05–1.65)*	1.25 (1.08–1.46)*	1.25 (1.09–1.45)*
PMW	1	1	1	1

^&^The results were generated by controlling gender, age, education level, marital status. *Confidence intervals statistically significant at the *p* < 0.05 level.

### 3.3 Correlates of mental health problems in different occupations

The results of significant associations in univariate logistic regressions and multivariable logistic regression in three occupations were shown in Table 4. Training experience was not included because the sample size of workers without training experience was too small. For all three occupations, the mental health problems were not associated with gender, marital status, having children younger than 18 years old, professional titles or previous experience in treating infectious diseases.

**Table 4:** Correlates of mental health problems in each occupation of medical workers.

Variables		Doctors		Nurses		PMWs	
		Crude OR (95%CI)	Adjusted OR (95%CI)	Crude OR (95%CI)	Adjusted OR (95%CI)	Crude OR (95%CI)	Adjusted OR (95%CI)
Age group	< 35	0.83 (0.65–1.07)	0.98 (0.72–1.33)				
	35–44	1.27 (1.01–1.59)*	1.31 (1.01–1.70)*				
	> 44	1	1				
Education level	College and lower	2.12 (1.37–3.28) *	2.56 (1.54–4.24)*			1.89 (1.01–3.52) *	2.8 (1.40–5.61)*
	Bachelor	1.35 (1.11–1.66) *	1.28 (1.01–1.62)*			1.94 (1.09–3.43) *	2.07 (1.10–3.90)*
	Master or PhD	1	1			1	
Living with family members	No	1.56 (1.27–1.92) *	1.63 (1.26–2.12)*				
	Yes	1	1		1		
Designated hospital for treating COVID-19	Yes	1.45 (1.20–1.76) *	1.20 (0.95–1.52)	1.27 (1.1–1.48) *	1.22 (1.02–1.45)*		
	No	1	1	1	1		
Department	High-risk	1.45 (1.20–1.76) *	1.07 (0.82–1.39)	1.20 (1.03–1.39) *	0.96 (0.80–1.15)	2.09 (1.25–3.49) *	1.44 (0.81–2.59)
	Low-risk	1	1	1	1	1	
Experience in treating COVID-19	Yes	1.42 (1.66–1.73) *	1.00 (0.75–1.33)				
	No	1					
COVID-19 event exposures	Yes	1.45 (1.13–1.84) *	1.15 (0.86–1.52)	1.49 (1.14–1.96) *	1.34 (1.01–1.83)*		
	No	1	1	1	1		
Overworked	Yes	3.23 (2.64–3.96)*	1.45 (1.06–1.98)*	3.22 (2.69–3.86)*	1.65 (1.31–2.07)*	2.86 (2.04–4.01)*	1.67 (1.07–2.61)*
	No	1	1	1	1	1	1
Enough rest time	No	3.3 (2.69–4.06)*	1.73 (1.26–2.36)*	3.53 (2.96–4.22)*	2.00 (1.61–2.48)*	5.83 (3.63–9.36)*	2.04 (1.32–3.14)*
	Yes	1	1	1	1	1	1
Adequate PPE	Partly adequate	1.16 (0.86–1.55)	0.75 (0.53–1.04)	1.31 (1.06–1.63)*	0.97 (0.77–1.23)		
	Not adequate	1.97 (1.46–2.64)*	0.85 (0.60–1.20)	2.12 (1.71–2.64)*	1.05 (0.82–1.34)		
	Very adequate	1	1	1	1		
Care from leaders	No	2.36 (1.94–2.86)*	1.03 (0.79–1.34)	2.28 (1.96–2.67)*	1.10 (0.89–1.35)	2.59 (1.89–3.56)*	1.54 (1.02–2.34)*
	Yes	1	1	1	1	1	1
Colleagues’ support	Most of the time	2.3 (1.86–2.86)*	1.32 (1.01–1.74)*	1.80 (1.53–2.12)*	1.08 (0.88–1.33)	1.89 (1.34–2.68)*	1.01 (0.65–1.57)
	Hard to say or no	6.71 (4.89–9.2)*	2.34 (1.55–3.54)*	5.00 (3.84–6.52)*	1.72 (1.22–2.42)*	5.70 (3.49–9.29)*	2.18 (1.14–4.15)*
	always	1	1	1	1	1	1
Family support	Partly support	2.22 (1.80–2.74)*	1.45 (1.13–1.86)*	1.80 (1.52–2.12)*	1.20 (0.98–1.46)	2.1 (1.48–2.97)*	1.21 (0.79–1.85)
	Not support	6.98 (4.68–10.41)*	3.21 (2.01–5.12)*	4.18 (3.09–5.64)*	1.74 (1.23–2.46)*	4.5 (2.36–8.58)*	1.81 (0.87–3.77)
	Totally support	1	1	1	1	1	1
Previous mental health status	Good	1.81 (1.45–2.27)*	1.24 (0.92–1.67)*	1.64 (1.38–1.94)*	1.44 (1.13–1.83)*	1.62 (1.13–2.30)*	1.22 (0.74–2.00)
	Just so or poor	6.70 (5.04–8.90)*	3.36 (2.28–4.95)*	5.98 (4.77–7.50)*	3.86 (2.82–5.28)*	5.83 (3.63–9.36)*	2.99 (1.54–5.78)*
	Very good	1	1	1	1	1	1
Previous physical health status	Good	1.82 (1.45–2.30)*	1.23 (0.90–1.67)	1.44 (1.21–1.71)*	0.85 (0.68–1.09)	1.80 (1.25–2.60)*	1.15 (0.70–1.88)
	Just so or poor	4.67 (3.57–6.11)*	1.61 (1.10–2.36)*	3.40 (2.77–4.17)*	0.95 (0.72–1.28)	3.52 (2.30–5.40)*	1.29 (0.71–2.33)
	Very good	1	1	1	1	1	1

*Confidence intervals statistically significant at the *p* < 0.05 level.

In the multivariable regression, doctors aged 35–44 years (OR = 1.31; 95%CI: 1.01–1.70), with a college certificate or lower (OR = 2.56; 95%CI: 1.54–4.24) or bachelor’s degree (OR = 1.28; 95%CI: 1.01–1.62), not living with family members (OR = 1.63; 95%CI:1.26–2.12), being overworked (OR = 1.45; 95%CI:1.06–1.98), not having enough time to rest (OR = 1.73; 95%CI:1.26–2.36), not always having support from colleagues (most of the time: OR = 1.32; 95%CI:1.01–1.74; hard to say or no: OR = 2.34; 95%CI:1.55–3.54), less family’s support (partly support: OR = 1.45; 95%CI:1.13–1.86; no support: OR = 3.21; 95%CI:2.01–5.12), poor previous mental health status (OR = 3.36; 95%CI:2.28–4.95) and poor previous physical health status (OR = 1.61; 95%CI:1.10–2.36) were more likely to have more mental health problems.

Nurses working in a designated institution for COVID-19 treatment (OR = 1.22; 95%CI:1.02–1.45), positive COVID-19 event exposures (OR = 1.34; 95%CI:1.01–1.83), being overworked (OR = 1.65; 95%CI:1.31–2.07), not having enough time to rest (OR = 2.00; 95%CI:1.61–2.48), less support from colleagues (OR = 1.22; 95%CI:1.02–1.45), less family support (OR = 1.74; 95%CI:1.23–2.46), and poor previous mental health status (OR = 3.86; 95%CI:2.82–5.28) were likely to have a higher prevalence of mental health problems than the contrast group.

PMWs with a college certificate or lower (OR = 2.80; 95%CI:1.40–5.61) or bachelor’s degree (OR = 2.07; 95%CI:1.10–3.90), working in a high-risk department (OR = 1.22; 95%CI:1.02–1.45), being overworked (OR = 1.67; 95%CI:1.07–2.61), not having enough time to rest (OR = 2.04; 95%CI:1.31–3.14), not having enough care from leaders (OR = 1.54; 95%CI:1.02–2.34), less support from colleagues (OR = 2.18; 95%CI:1.14–4.15), and poor previous mental health status (OR = 2.99; 95%CI:1.54–5.78) were likely to have a higher prevalence of mental health problems than the contrast group.

## 4 Discussion

This study is a large sample online survey of the prevalence and correlates of mental health problems among three occupations of medical workers at the initial stage of the COVID-19 pandemic. At that time, most medical workers in China had been engaged in high-intensity work for over 2 months without effective treatments and vaccines, which may persist for an extended period. Previous studies indicated that an approximately 26% rise in depressive and anxiety symptoms was observed among the general population worldwide since the onset of the pandemic (33), showing that the mental health status of the public was worrying. Medical workers faced a more dangerous and vulnerable environment than the general population during the COVID-19 pandemic (34), which may have a negative impact on their emotional state and even lead to mental health problems. From our survey, the prevalence of any mental health problems varied among different occupations, with doctors having a prevalence rate of 32.9 and 43.6% in nurses; that of anxiety in three occupations was from 27.9% in doctors to 39.1% in nurses; and that of depression was from 13.7% in PMW to 17.4% in nurses. Nurses exhibited a higher prevalence of depression, anxiety, and any mental health problems compared to doctors and PMWs. Doctors experienced more significant psychological pain than PMWs. The prevalence of mental health problems among nurses, doctors, and PMWs reported in the current study is consistent with the range reported in other regions of China, and some meta-analyses of studies conducted in other countries that of anxiety symptoms among medical workers during the COVID-19 varied from 11.6 to 44.6% (7, 35, 36), and from 12.2 to 43.6% in the case of depression symptoms (35, 37). Moreover, the current study showed that mental health problems in different occupations were correlated with different factors.

### 4.1 Sociodemographic factors

A previous study found that women reported more severe symptoms of depression, anxiety, and distress (7). However, there was no correlation with gender across the three occupations in our study. This may result from the number of female medical workers involved. There were 76.7% females in the previous, while in our study, 80.8% of participants were women. In addition, the previous study mainly consisted of physicians and nurses (7, 11); we included paramedical workers, which may be another potential reason. The study found a significant correlation between educational level and mental health in doctors and PMWs, indicating that individuals with lower educational levels were prone to experience mental health problems. In this study, doctors had the highest percentage of individuals with master’s or PhD degrees, followed by PMWs and then nurses. Even though more than 98% of medical workers were trained with COVID-19, individuals with higher education were less likely to be influenced by the outbreak, which may be attributed to the potential impact of higher education on enhancing individuals’ understanding of psychological distress issues. Consequently, individuals with higher education may be more likely to take positive measures to prevent related symptoms (38). The correlation between age and mental problems was shown in doctors, indicating that younger doctors had more emotional problems than older people. This was in line with the results of previous studies that have shown that younger medical workers had higher stress and depressive symptoms than older workers (18, 39). However, this relationship did not exist in both nurses and PMWs.

### 4.2 Working status and associated factors

Working status during the outbreak was an essential in impacting the mental health of medical workers (9, 10). Being overworked and not having enough time to rest were related to mental health problems in all three occupations, which was in line with previous studies indicating that due to the heavy workloads and hazardous working environments, healthcare workers were at high risk of psychological stress (6, 10, 39, 40). Therefore, it is necessary to provide more healthy and safe working conditions to healthcare workers. Although wearing personal protective equipment was the most effective and common way to protect medical workers from being infected in the context of lacking any clear treatments, our results indicated that personal protective equipment was not the influencing factor of mental health problems in all three occupations, which was inconsistent with previous studies which indicated that inadequate PPE was associated with psychological stress and depressive symptoms (41). The greater accessibility of PPE compared with general people may explain this difference. A study showed that the general public who have less access to PPE could contribute to a higher prevalence of anxiety and depression than that of frontline medical workers (35). Another unexpected result was that the working department was not related to mental health problems in doctors, nurses, and PMWs. This result disagreed with a previous study conducted during the SARS (15); the difference may be due to the following reason. The average incubation period for COVID-19 was 14 days; it was uncertain whether the patients they treated in low-risk departments were infected with COVID-19 during the incubation period (11). Consequently, medical workers in high-risk or low-risk departments had to be worried about the infectious risks.

### 4.3 Social support factors

Social support is also a key for medical workers to cope with psychological symptoms (15). The findings presented in the current study indicated that individuals having less support from others had a high prevalence of mental health problems. For doctors and nurses, support from colleagues and family, but not from their supervisor or head of a department, could reduce mental health problems. This finding aligned with the study conducted in Singapore (42), which revealed that doctors and nurses who encountered psychiatric symptoms found the support from their supervisor or head of the department unhelpful. However, they reported less emotional distress when they received support from their colleagues. For PMWs, individuals who did not receive enough support from their supervisor or head of a department reported more mental problems than those who received. It may be that the work of PMWs is more relevant to the authorities than other occupations during the outbreak. Those results were consistent with prior research, which showed that receiving support from family, friends, and colleagues may serve as a protective factor for mental health during infectious disease outbreaks (43, 44). Furthermore, our study provided more evidence that medical workers in different occupations may need support from different sources.

### 4.4 COVID-19 event exposures

For nurses but not for doctors and PMWs, their mental health status was associated with their experience of COVID-19 event exposures. In this sample, quarantining was discovered to be a strong predictor of a high level of mental health issues, which is consistent with earlier studies examining the psychological impact of quarantining on both the general population and front-line healthcare staff involved in combating an outbreak (45, 46). Overall, researchers point to a wide-ranging, substantial, and prolonged psychological impact of quarantine (47). Furthermore, another study found a robust correlation between the experience of being in quarantine and the severity of depressive symptoms, even 3 years after the isolation measures were terminated (48).

### 4.5 Previous mental health status

Previous mental health status was relevant to mental health during the outbreak in all three vocations examined in this study. The finding was line with a prior study, which indicated that health workers with poor or fair health conditions had higher stress and mental problems (49). Poor previous physical health status was a risk factor for mental health in doctors but not in nurses and PMWs.

## 5 Limitations

Some limitations should be acknowledged. First, the voluntary nature of participation may introduce self-selection sampling bias, potentially influencing the prevalence of mental health problems. Second, the sample predominantly consisted of participants from provinces and cities outside Hubei, where the epidemic was most severe in China, limiting the generalizability of the findings. Third, due to the cross-sectional design, causal relationships cannot be determined. Even though we found different correlates in the three occupations, we did not investigate the underlying mechanism as to why some factors were unique to one occupation. Further research is needed to understand the correlations. Fourth, due to the time restriction of the current survey, some other factors like personality (50, 51), coping strategies (18, 52), or parental information (50) were not collected in the current survey. Despite these limitations, this study examined the effects of COVID-19 on different medical occupations and identified associated factors during the peak of the outbreak.

## 6 Conclusion

In summary, this study aims to investigate the prevalence and associations of the mental health problems in doctors, nurses, and PMWs during the peak of the COVID-19 in China. The finding shows that the three kinds of medical workers reported high rates of any mental health problems. Three occupations shared some main factors association with mental health problems, including being overworked, not having enough time to rest, support from colleagues, and previous mental health status. And each occupation has their own specific factors. This indicated that measures taken to promote the mental health of medical workers and prepare them for future disease outbreaks should consider the different needs of medical workers in various occupations.

## Data availability statement

The raw data supporting the conclusions of this article will be made available by the authors, without undue reservation.

## Ethics statement

The Ethical Committee of Beijing Ditan Hospital Capital Medical University (BJDTH) has granted approval for this survey [JINGDILUNKE (2020)-(012)-01]. The studies were conducted in accordance with the local legislation and institutional requirements. The ethics committee/institutional review board waived the requirement of written informed consent for participation from the participants or the participants’ legal guardians/next of kin because writing informed consent was not required of any participant in this web-based anonymous survey, as their participation was entirely voluntary.

## Author contributions

QY: Conceptualization, Writing – original draft, Investigation, Writing – review & editing. XH: Conceptualization, Validation, Writing – review & editing. TZ: Data curation, Writing – original draft, Methodology, Writing – review & editing. HY: Investigation, Writing – original draft, Formal analysis, Writing – review & editing. BH: Formal analysis, Writing – review & editing. CM: Software, Writing – review & editing. YW: Software, Writing – review & editing. HC: Methodology, Writing – review & editing. JW: Writing – review & editing. YH: Supervision, Writing – review & editing. RJ: Resources, Writing – review & editing. ZL: Supervision, Writing – review & editing. GX: Resources, Writing – review & editing.

## Footnotes

^1^ https://www.wjx.cn/
